# The Sphkl/SlP pathway regulates angiogenesis via NOS/NO synthesis following cerebral ischemia‐reperfusion

**DOI:** 10.1111/cns.13275

**Published:** 2019-12-08

**Authors:** Man-Hua Lv, Shi Li, Yong‐Jia Jiang, Wei Zhang

**Affiliations:** ^1^ Department of Neurology The First Affiliated Hospital of Harbin Medical University Harbin China; ^2^ Department of Neurology The Central Hospital of Wuhan Tongji Medical College Huazhong University of Science and Technology Wuhan China

**Keywords:** cerebral ischemia‐reperfusion, endothelial cell, nitric oxide, sphingosine kinase 1, sphingosine‐1‐phosphate

## Abstract

**Aims:**

Sphingosine kinase 1 (Sphk1) and the signaling molecule sphingosine‐1‐phosphate (S1P) are known to be key regulators of a variety of important biological processes, such as neovascularization. Nitric oxide (NO) is also known to play a role in vasoactive properties, whether Sphk1/S1P signaling is able to alter angiogenesis in the context of cerebral ischemia‐reperfusion injury (IRI), and whether such activity is linked with NO production, however, remains uncertain.

**Methods:**

We used immunofluorescence to detect the expression of Sphk1 and NOS in cerebral epithelial cells (EC) after IR or oxygen‐glucose deprivation (OGDR). Western blotting was used to detect the Sphk1 and NOS protein levels in brain tissues or HBMECs. Adenovirus transfection was used to inhibit Sphk1 and NOS. An NO kit was used to detect NO contents in brain tissues and epithelial cells. Tube formation assays were conducted to measure angiogenesis.

**Results:**

We determined that EC used in a model of cerebral IRI expressed Sphk1, and that inhibiting this expression led to decreased expression of two isoforms of NO synthase (eNOS and iNOS), as well as to decrease neovascularization density and NO production following injury. In HBMECs, knocking down Sphk1 markedly reduced NO production owing to reduced eNOS activity, and inhibiting eNOS directly similarly decreased NO production in a manner which could be reversed via exogenously treating cells with S1P. We further found that knocking down Sphk1 reduced HBMEC eNOS expression, in addition to decreasing the adhesion, migration, and tube formation abilities of these cells under OGDR conditions.

**Conclusions:**

Based on these results, we therefore postulate that Sphk1/S1P signaling is able to mediate angiogenesis following cerebral IRI via the regulation of eNOS activity and NO production. As such, targeting these pathways may potentially represent a novel means of improving patient prognosis in those suffering from cerebral IRI.

## 1 INTRODUCTION

Stroke remains the most common cause of death and disability worldwide,^1^ with cerebral ischemia‐reperfusion injuries (IRIs) having the potential to induce additional debilitating secondary damage. At present, there is a major lack of effective drugs for the treatment of ischemic stroke,^2^, ^3^ and treating the underlying damage using nerve repair‐based approaches is hampered by the underlying vascular dysfunction which can prevent neurogenesis in affected areas of the brain.^4^ As such, there is a clear need to develop novel therapeutic strategies which can promote angiogenesis, thereby facilitating more effective blood perfusion into injured areas of the brain following IRI, thus allowing for improved healing.^5^, ^6^, ^7^ Vasculogenesis is the process by which primitive mesodermal cells home to various tissues and differentiate into endothelial cells (ECs) to form the primary capillary network.

Nitric oxide (NO) is a key vasoactive signaling molecule which can modulate local blood flow via promoting vasodilation and reducing cerebral vascular resistance.^8^, ^9^ The NO signaling molecule is a highly reactive and short‐lived small molecule that can be produced by three forms of NO synthase (NOS) enzymes in specific contexts, with ECs and perivascular nitrergic neurons being primary sources of NO production.^10^ When release in the brain, NO can be both beneficial and harmful depending on the specific context, with factors including the NOS isoform induced, the cellular source of NO, and the time relative to IRI being critical for ultimately determining its relative harm or benefit. For example, immediately following ischemia of the brain, eNOS activity has been shown to be protective and promotes angiogenesis, maintains vascular endothelial function, and regulates vascular tone, whereas neuronal NOS (nNOS) and inducible NOS (iNOS)‐mediated NO production have been associated with subsequent brain damage.^10^, ^11^, ^12^, ^13^

Sphingosine‐1‐phosphate (S1P) is a sphingolipid metabolite that plays a wide range of biological activities in processes such as cell proliferation, migration, survival, and, of interest for this study, angiogenesis.^14^, ^15^ Sphk1/S1P signaling has previously been shown to regulate microglial IL‐17A production via TRAF2/NF‐κB signaling in microglial cells, thereby inducing the apoptotic death of neurons in the context of IRI.^16^, ^17^ The enzyme sphingosine kinase (SphK) is responsible for phosphorylating sphingosine in order to produce the angiogenic S1P, which is then secreted from cells whereupon it can regulate the actions of ECs.^15^ Mammalian cells have two primary SphK isoforms, with Sphk1 being the primary isoform relevant in ECs.^18^, ^19^ The SphK1/S1P signaling axis is thought to be of particular importance in processes regulated by S1P, in addition to its importance in the context of atherosclerosis, inflammation, and oncogenesis.^20^, ^21^, ^22^, ^23^ Exactly how and whether SphK/S1P signaling regulates angiogenesis, however, remains to be fully established.

When an ischemic stroke occurs, the subsequent diastolic and vasoconstrictive activity in the brain is tightly linked with eventual patient prognosis. The S1P receptor, which is induced in cerebral microvessels early after I/R injury, potently regulates brain endothelium responses to ischemic and inflammatory injury.^24^ In addition, previous work in HUVEC cells has shown that knocking down Sphk1 can markedly impair their proliferation and migration.^25^ How endothelial Sphk1/S1P signaling impacts stroke prognosis via neovascularization and collateral establishment, however, remain uncertain.

We hypothesize that endothelial SphK1/S1P signaling can increase NOS activity following cerebral ischemia, leading to dilation of local vasculature and thus reducing leukocyte adhesion and platelet aggregation, leading to improve patient prognosis. NO synthesis has not been adequately studied in the context of the role of Sphk1/S1P on cerebral ischemia prognosis, and thus, our research has the potential to identify novel therapeutic avenues for future treatment development.

## 2 MATERIALS AND METHODS

### 2.1 Animals

Male Wistar rats (240‐260 g) from the Experimental Animal Center of the second affiliated hospital of Harbin Medical University were housed under standard conditions in a temperature and humidity‐controlled environment with a 12 hours light/dark cycle and with free food and water access. Animals were allowed to acclimate for 1 week prior to use. The School of Medical Science, Harbin Medical University approved all animal protocols, and all efforts were made to minimize animal utilization and suffering where possible.

### 2.2 Reagents and antibodies

Rabbit anti‐rat eNOS, rabbit anti‐rat iNOS, rabbit anti‐rat nNOS, rabbit anti‐rat Sphk1, mouse anti‐rat CD31, rabbit anti‐rat CD31, rabbit anti‐rat ERG, human anti‐rat Sphk1, and human anti‐rat iNOS were purchased from Abcam. Fluorescently conjugated secondary anti‐rabbit and anti‐mouse IgG were from Invitrogen. Sigma was the source of S1P, while an NO assay kit was from Nanjing Jiancheng Bioengineering Institute.

### 2.3 Focal cerebral ischemia‐reperfusion model

We utilized a rat model of middle cerebral artery occlusion (MCAO) via thread embolization as described previously, with slight adjustments.^26^ Briefly, animals were given 10% chloral hydrate solution (350 mg/kg) i.p. to induce anesthesia, after which the neck was surgically opened to reveal the common carotid artery (CCA), with the distal end of the external carotid artery (ECA) being ligated as close to the head as possible. A small V shape was then cut into the ECA at the distal end, through which a MCAO thread bolt was then inserted. The ECA was manipulated to align with the ICA prior to loosening the ICA clamp and slowly inserting the thread into the ICA. Once blood flow had been blocked for a 2‐hour period, the embolic device was removed to the stump of the ECA so as to simulate IRI. As a control, sham rates were treated in the same manner but without any thrombus insertion. Experimental studies were conducted on these animals at 2, 6, 24, and 48 hours after operation.

### 2.4 Immunohistochemistry

Endothelial cells positive for the expression of Sphk1, iNOS, eNOS, and nNOS were identified in the context of cerebral IRI via double immunofluorescent staining. Formalin‐fixed paraffin‐embedded tissue sections from study animals were first de‐paraffinized using xylene, then treated with citric acid to promote antigen retrieval. Sections were washed thrice in PBS, blocked using serum for 1 hour, and then probed overnight with appropriate primary antibodies at 4°C. Samples were then washed and probed with appropriate fluorescently conjugated secondary antibodies for 1 hour at room temperature. An Olympus FV300 confocal microscope (200×) was used for sample imaging, with the Image‐Pro Plus 6.0 software used for determining protein amounts based on mean optical density (OD) values. A total of 4‐5 random fields were assessed in different regions of each cortex sample, with average OD values reported.

### 2.5 Western blotting

Total eNOS, nNOS, and iNOS levels were assessed via Western blotting at appropriate time points following MACO or OGDR. BCA assay was used to determine total protein levels in available samples, after which proteins were separated on 10%‐12% SDS‐PAGE gels, and then transferred to cellulose acetate membranes. Resultant blots were blocked with 5% nonfat milk at room temperature for 2 hours, after which they were probed overnight with appropriate primary antibodies at 4°C. After washing, blots were then probed with appropriate fluorescently labeled secondary antibodies for 1 hour, and relative protein levels were then assessed with an Odyssey 3.0 infrared imaging system, with β‐actin bands used to normalize protein loading content between samples.

### 2.6 Sphk1 suppression through adenoviral interference

Animals were anesthetized with 3.5% chloral hydrate (350 mg/kg) and placed in a stereotactic device. Animals were then administered an Sphk1‐targeting adenovirus (2 μg/μL) in the right ventricle 1.0 mm behind the anterior iliac crest, 2.0 mm to the side of the stereotactic instrument, under the guidance of the midline and the 3.5 mm ventral side of the skull surface. Two weeks following this injection, MCAO modeling was performed.

### 2.7 Angiography

At appropriate time points, animals were euthanized and PBS was perfused through the left ventricle until there was negligible blood detectable in the atrial vent. MICROFIL compound (Flow Tech Inc) was then prepared by adding an appropriate curing agent, after which this compound was infused via the ventricular cannula until the compound was observed to freely flow out of the atrial vent. At this point, clamps were used to close both the ventricular and atrial cannulas, and animals were moved to 4°C overnight. The following day, brains were dissected from the skull, keeping the dura mater intact, and were added to a 4.5% formalin solution for 24 hours prior to micro‐CT examination.

### 2.8 Injection of S1P

The dosing and treatment schedule of S1P injection was based on previously published reports.^27^, ^28^ S1P was dissolved in 0.9% saline solution. Intraperitoneal injection of S1P (0.05, 0.08, and 0.1 mg/kg) was performed 24 hours prior to operation, with the remaining control animals receiving an equivalent saline vehicle injection.

### 2.9 HBMEC culture

Human brain microvascular endothelial cells (HBMECs) were purchased from ScienCell and were cultured in fetal bovine serum in DMEM medium at 37°C in a 5% CO2 incubator. Cells between the 6th and 9th passages were used for experimentation, with endothelial phenotypes being confirmed based on positive CD31 expression and a lack of CD34/CD133 expression.

### 2.10 Adenovirus infection

An AdEasy Adenoviral Vector Systemby GenePharma Corporation. Was used to generate shRNA‐bearing adenovirus. Briefly, the Ad‐Sphkl‐shRNA, Ad‐eNOS‐shRNA, and Ad‐iNOS‐shRNA were cloned into the pShuttle‐CMV vector, which was then recombined into the pAdeasy‐1 adenovirus vector to generate the virus. Once generated, HBMECs were infected with these adenovirus; at an MOI = 100 for 24 hours, after which media were changed and cells were incubated for 24 hours further prior to reinfection under the same conditions.

### 2.11 Real‐time PCR (RT‐PCR)

An RNA isolation kit (BioTeke) was used for total HBMEC RNA isolation, with RNA being used for cDNA synthesis with a PrimeScript cDNA synthesis kit (BioTeke). Gene expression was then assessed via RT‐PCR with UltraSYBR (BioTeke) on an Exicycler 96 RT‐PCR platform (Bioneer). Primers were synthesized and validated by Bioneer biotechnology. Gene‐specific primers used as follows: Sphk1: forward, CGCTCTGGTGGTCATGTCTG and revised, GCAATAGCGTGCAGTTGGTC; β‐actin: forward, CACTGTGCCCATCTACGAGG and revised, TAATGTCACGCACGATTTCC.

### 2.12 Oxygen‐glucose deprivation reperfusion (OGDR) treatment

Cultured HBMECs were used to model OGDR conditions. To accomplish this, normal HBMEC growth media was exchanged for Earle's balances salt solution with no added glucose, and cells were transferred into a low‐oxygen (95% N2/3% CO2/2%O2) 37°C incubator for 2 hours. As a control, cells were cultured under normoxic conditions in Earle's balanced salt solution supplemented with 10 mmol/L glucose in parallel. OGD was then terminated via returning cells to normoxic condition so as to initiate reperfusion.

### 2.13 Cell stimulation

HBMECs were treated via addition of exogenous S1P (10 nmol/L) prior to OGDR in certain experiments, with control cells cultured in parallel without drug treatment. At appropriate time points, cells and/or supernatants were collected for analysis.

### 2.14 Endothelial tube formation and cell migration assays

To measure angiogenesis, a tube formation assay was conducted. Briefly, following experimental treatment cells were resuspended in fresh complete culture media and were plated in 96‐well Matrigel cell culture plates (10^4^ cells/well). Cells were then grown for 12 hours in normoxic or hypoxic incubators and were then imaged via inverted phase contrast microscope (100×). HBMECs which had been adenovirally infected for 24 hours were divided into four groups, and were treated for 1 hour with 1 µg/mL mitomycin C. A 200 µL pipette tip was then used to wound the cell monolayer, and cell migration into the wound site was recorded via microscopic imaging while cells were grown in serum‐free medium under hypoxic or normoxic conditions.

### 2.15 NO measurement

As NO metabolism ultimately leads to nitrite production, NO content was measured via quantifying nitrate and nitrite levels in samples. To achieve this, cadmium was used to covert nitrate to nitrite, after which Griess’ reaction was conducted, and absorption at 540 nm was measured with a microplate reader to determine NO content in a given sample.

### 2.16 Statistical analysis

All experiments were conducted thrice, and data are means ± standard deviation. SPSS v22.0 was used for statistical testing, with single‐factor ANOVAs and Student's *t* tests used for comparisons. *P* < .05 was the significance threshold.

## 3 RESULTS

### 3.1 Cerebral IRI induces endothelial Sphk1 expression

**Figure 1:**
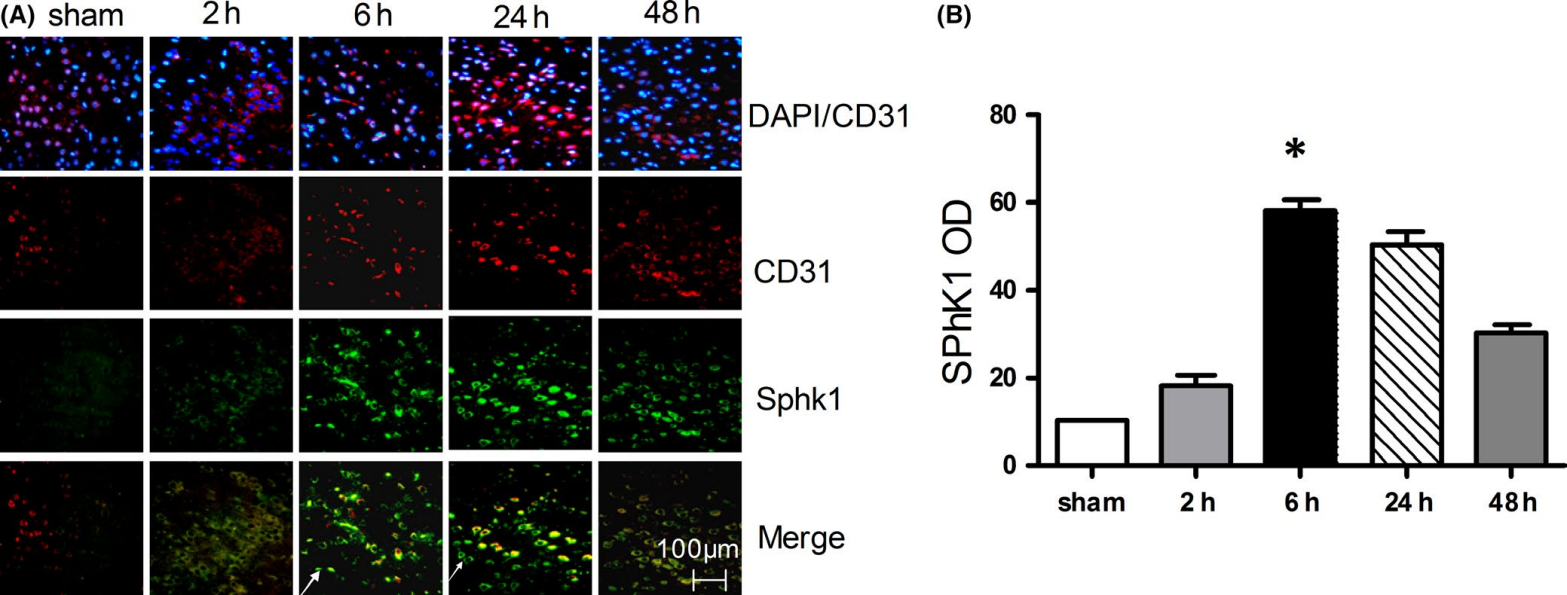
Sphk1 expression in a model of IRI. A, At 2, 6, 24, and 48 h following IRI modeling, brain tissue was stained for Sphk1 (green) and endothelial cells marker CD31 (red) and assessed by confocal microscopy. White arrows represent endothelial cells negative for Sphk1. B, Sphk1 OD measurements indicated a gradual induction over time after IRI. Data are means ± SD (n = 5). *P < .05 vs control

We first assessed the expression of Sphk1 in endothelial cells in the context of cerebral IRI by staining rat cerebral endothelial cells for this enzyme following induction of a MCAO model designed to simulate IRI. We determined that there was substantial expression of Sphk1 in the peri‐infarct cortex at 2, 6, 24, and 48 hours following IRI, with Sphk1‐positive cells also being CD31‐positive, indicating their status as endothelial cells (Figure 1A). We also observed CD31‐negative cell Sphk1 expression, as Sphk1 is also expressed in other nerve cells such as microglia (white arrow).^29^ In contrast, Sphk1 expression was not detectable in sham‐operated rats. Maximal relative Sphk1 expression in ECs was evident at 6 hours (59.67 ± 0.2; *P* < .05) following IRI, gradually decreasing by 48 hours (Figure 1B). This suggests that partial Sphk1 induction occurs in cerebral endothelial cells following IRI.

### 3.2 Endothelial cells induce eNOS and iNOS during IRI

**Figure 2:**
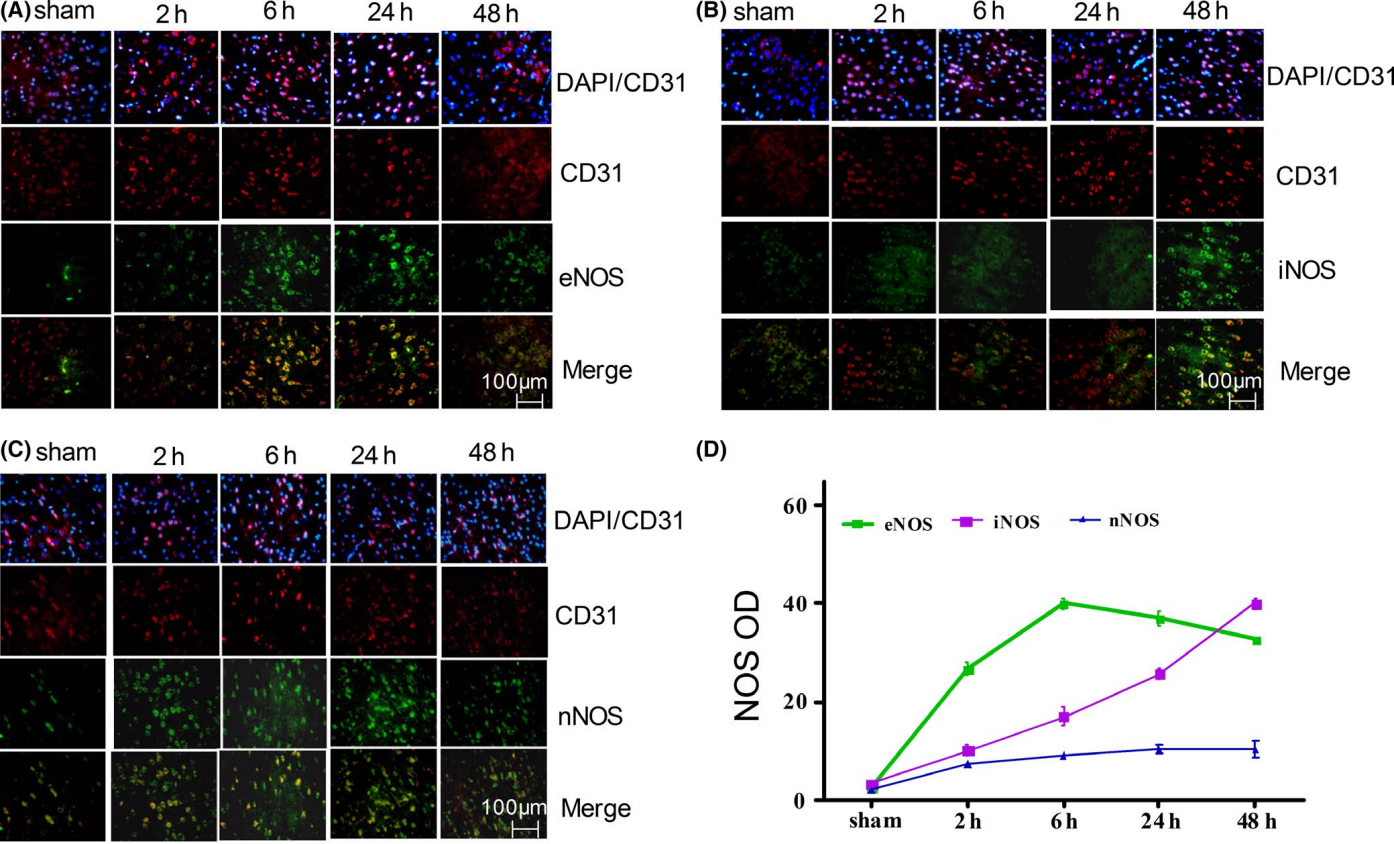
Induction of eNOS, iNOS, and nNOS in the brain after IRI. A–C, Tissue sections were stained for eNOS/ iNOS/ nNOS (green) and endothelial markers CD31 (red) at 2, 6, 24 at 48 h following IRI and were assessed by confocal microscopy. D, OD measurements indicated a gradual induction of eNOS/iNOS/nNOS after IRI. Data are means ± SD (n = 5). *P < .05 vs control

We next stained rat brain sections to assess endothelial expression of eNOS, nNOS, and iNOS at 2, 6, 24, and 48 hours following IRI. (Figure 2A‐C). Clear eNOS staining was evident within the peri‐infarct region at 6 hours postinjury, with these levels slowly falling by 48 hours (Figure 2A). At 2 hours postinjury, iNOS expression began to rise before reaching a maximum after 48 hours (Figure 2B), whereas maximal nNOS expression was evident at 6 hours postinjury and was largely absent in endothelial cells (Figure 2C). Indeed, consistent with these visual observations, maximal eNOS expression was evident at 6 hours (40.33 ± 0.23; *P* < .05), while that of iNOS was detectable at 48 hours (41.33 ± 0. 3; *P* < .05; Figure 2D). This suggests that endothelial cells rapidly induce eNOS following ischemia, while inducing iNOS at later time points.

### 3.3 Suppression of Sphk1 inhibits the induction of eNOS, iNOS, and neovascularization following IRI

**Figure 3:**
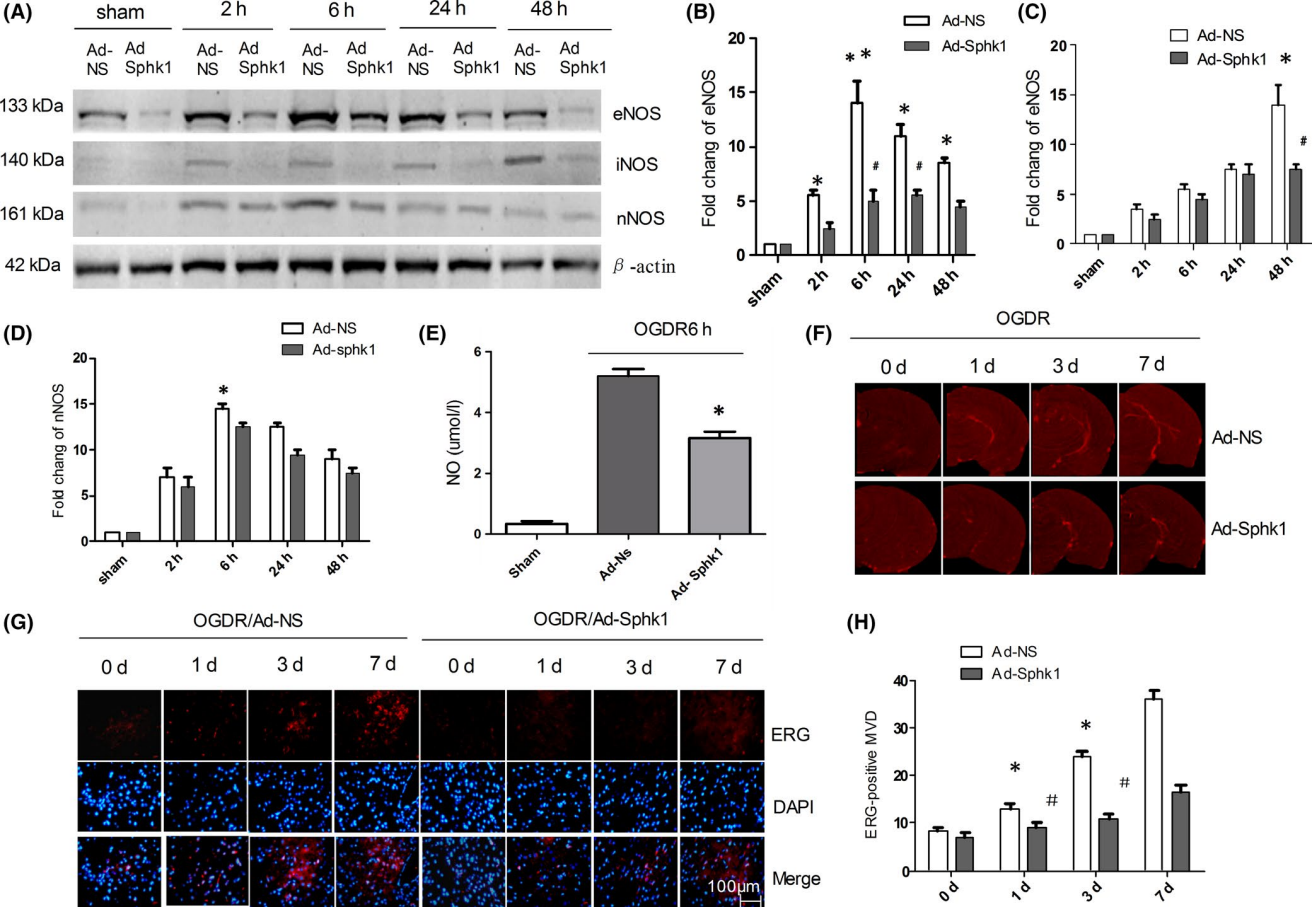
Sphk1 knockdown decreases cerebral eNOS and iNOS expression after IRI. A‐D, eNOS, iNOS, and nNOS levels were measured by Western blotting in the brains of IRI model animals, with β‐actin used for normalization. E, NO production was measured in the brains of IRI model animals infected with Ad‐NS or Ad‐sphk1. F, Detection of neovascularization in the infarcted cortex by micro‐CT at 1, 3, and 7 d after I/R in animals infected with Ad‐NS or Ad‐sphk1. G, H, Immunofluorescence staining of ERG‐positive MVD around the infarction in the cortex at 1, 3, and 7 d after I/R in Ad‐NS or Ad‐sphk1 infected animals. Three fields of view were measured in three different sections. Data are means ± SD. *P < .05 vs control. **P < .01, vs Sham group; #*P* < .05, ##*P* < .01 vs Ad‐NS group

We next began to assess the importance of Sphk1/S1P signaling in angiogenesis and collateral establishment in the context of IRI by treating IRI model rats with shRNA adenoviral vectors to stably knockdown Sphk1. For this study, the expression of eNOS and nNOS increased to a peak at 6 hours (14 ± 0. 22; *P* < .01 14.5 ± 0. 13; *P* < .05); at 48 hours, the expression of iNOS increased significantly (14.4 ± 0.15; *P* < .05; Figure 3A‐D). In Ad‐Sphk1 group, Western blotting revealed that Ad‐Sphk1 markedly reduced both eNOS and iNOS expression following IRI (5.0 ± 0. 1; 7.5 ± 0.18; *P* < .05; Figure 3A‐C), whereas nNOS was not affected (*P* > .05; Figure 3A,D). NO content measurements also revealed that the Ad‐Sphk1 group had significantly lowered NO levels than did the Ad‐NS group at 6 hours post‐I/R (3.166 ± 0. 21; *P* < .05; Figure 3E). New blood vessels that form after focal ischemia appear by days 4‐7 in the peri‐infarct regions.^30^ We therefore examined our samples at days 1, 3, and 7 postinfarction. Micro‐CT angiography confirmed that after Ad‐Sphk1 inhibition, numbers of collateral vessels in the infarcted area decreased relative to the Ad‐NS group (Figure 3F). The endothelial transcription factor ERG is expressed in endothelial cells and regulates multiple pathways involved in vascular homeostasis, development, stability, and angiogenesis.^31^, ^32^, ^33^ As such, we examined ERG expression as a marker of neovascularization. Relative to the Ad‐NS group, MVD decreased significantly at 3 and 7 days post‐I/R (*P* < .05) in the Ad‐Sphk1 group, whereas differences were not significant at day 1 (Figure 3G,H). This suggests that in the context of cerebral IRI, Sphk1 is important for regulating angiogenesis and collateral vessel establishment.

### 3.4 S1P increases the induction of eNOS, iNOS, and NO production following IRI

**Figure 4:**
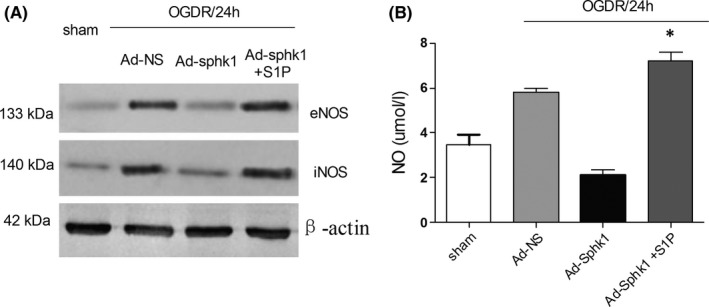
Effects of S1P on MACO rats. S1P was injected (0.1 mg/kg) i.p into rats 24 h prior to operation, with the remaining control animals receiving an equivalent saline vehicle injection. A, Induction of eNOS and iNOS was evaluated via Western blotting in animals dosed with or without S1P at 24 h following cerebral IR. B, NO production was measured by ELISA in the brains of IRI model animals transfected with Ad‐NS or Ad‐sphk1. Data are means ± SD. *P < .05 vs control. **P < .01 vs Sham group

S1P is known to regulate the proliferation, migration, survival, and angiogenesis of epithelial cells.^34^, ^35^, ^36^ Rats were injected i.p with different concentrations of S1P (0.05, 0.08, and 0.1 mg/kg), and after 24 hours, the optimal effective dose of S1P was assessed via ELISA. When the injection dose of S1P reached 0.1 mg/kg, the content of S1P in brain tissue is the most (6.2 ± 0.25; *P* < .01; Supplemental Figure S1). Western blotting was used to asses protein levels from animals dosed with or without S1P (0.1 mg/kg) following cerebral I/R for 24 hours. Induction of eNOS and iNOS was increased following injection of S1P in the Ad‐Sphk1 group compared to the Ad‐NS group, (*P* < .01, Figure 4A). Measurement of brain NO contents revealed that at 24 hours following I/R, the Ad‐Sphk1 group had significantly higher NO levels than the Ad‐NS group puls S1P (Figure 4B). This suggests that in the context of cerebral IRI, Sphk1/S1P is important for regulating the induction of eNOS and iNOS as well as NO production.

### 3.5 Sphk1 regulates eNOS/iNOS expression and NO production by HBMECs

**Figure 5:**
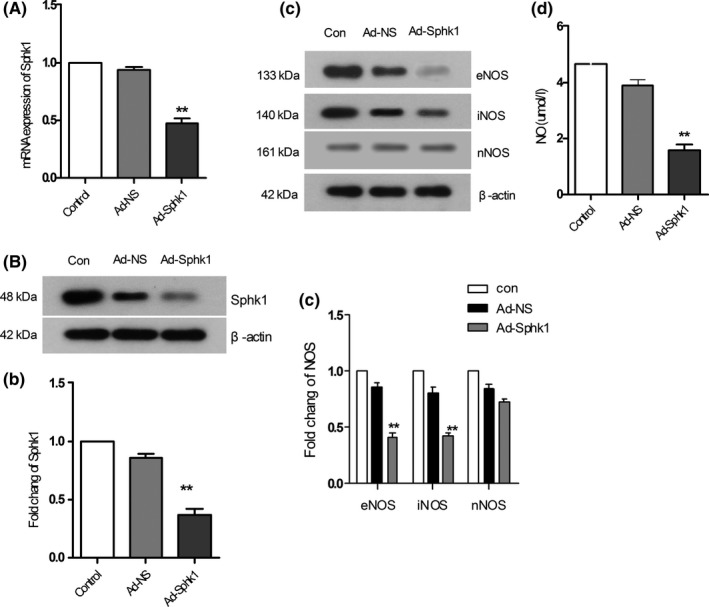
Effects of Sphk1 knockdown in HBMECs. A, RT‐PCR was used to assess HBMEC Sphk1 expression after control or Sphk1 adenovirus infection. B, b, Sphk1 protein levels in cells treated as in (A) as measured by Western blotting. C, eNOS, iNOS, and nNOS protein levels were assessed in HBMECs treated as in (B) by Western blotting. D, NO production of adenovirally infected HBMECs was measured. Data are means ± SD. *P < .05; **P < .01 vs control

To further confirm the role of Sphk1 in endothelial NO production, we next used shRNA adenoviral vectors to stably knock down Sphk1 in HBMECs, confirming reduced expression by real‐time PCR and Western blotting 24 hours postinfection (52% mRNA knockdown; 62% protein knockdown; Figure 5A,B,b). We then assessed the levels of eNOS, iNOS, and nNOS present in these cells, revealing that SphK1 knockdown resulted in decreased eNOS and iNOS expression (64% and 51% protein knockdown; *P* < .01; Figure 5C,c), without any significant effect on nNOS expression (Figure 5C,c). We further measured NO production by these cells, which similarly confirmed that Sphk1 knockdown inhibited NO production by HBMECs (*P* < .01; Figure 5D). This therefore confirms that Sphk1 is important for mediating iNOS and eNOS induction and consequent NO production by HBMECs.

### 3.6 Loss of eNOS and iNOS inhibits NO production, while exogenous S1P induces eNOS expression and NO production by HBMECs

**Figure 6:**
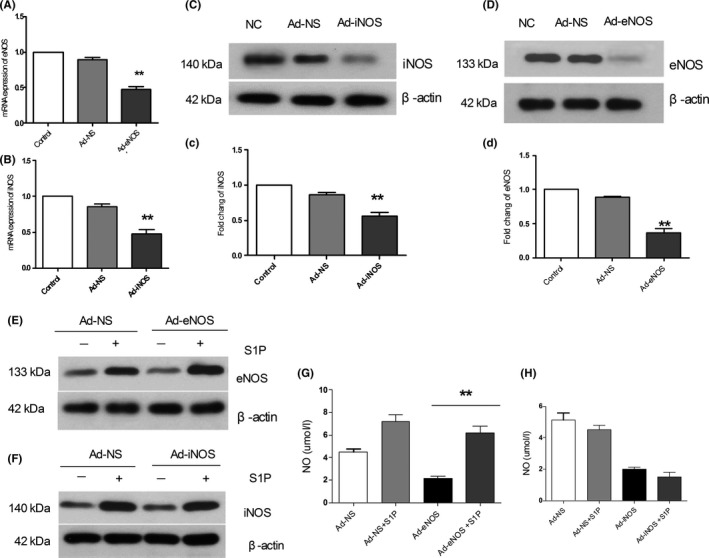
Effects of eNOS and iNOS knockdown in HBMECs. A, B, Relative eNOS/iNOS levels were assessed via RT‐PCR after respective knockdown with an adenoviral vector or control virus infection. C, D, eNOS/iNOS protein levels were assessed in cells infected with the indicated adenoviruses by Western blotting. E, F, HBMECs were cultured for 12 in serum‐free media with or without S1P, and then, eNOS/iNOS expression in cells infected with eNOS/iNOS‐specific adenoviruses or controls by Western blotting. G, H, Cells were treated as in (A/B), and then, NO production was measured. Data are means ± SD. *P < .05; **P < .01 vs control

Our results thus far have demonstrated that Sphk1 is important for NO production in endothelial cells, but which enzymes are essential for this process remain uncertain. We therefore infected cells with adenoviruses bearing shRNA constructs specific for eNOS or iNOS, confirming knockdown of these enzymes via real‐time PCR and Western blotting (*P* < .01; Figure 6A‐D). We therefore sought to assess whether S1P was able to directly affect NO production by treating cells with 10 nmol/L exogenous S1P for 12 hours and then measuring eNOS and iNOS expression, as well as NO production. We found that S1P treatment significantly induced both iNOS and eNOS even in cells in which Sphk1 had been knocked down (Figure 6E,F). S1P further enhanced eNOS, NO production in HBMECs (6.3 ± 0.2; *P* < .01; Figure 6G), but not iNOS (*P* > .01; Figure 6H). This therefore suggests that the Sphk1/S1P pathway directly regulates eNOS induction, thereby controlling NO production.

### 3.7 Loss of Sphk1 impairs eNOS induction and angiogenesis under OGDR conditions

**Figure 7:**
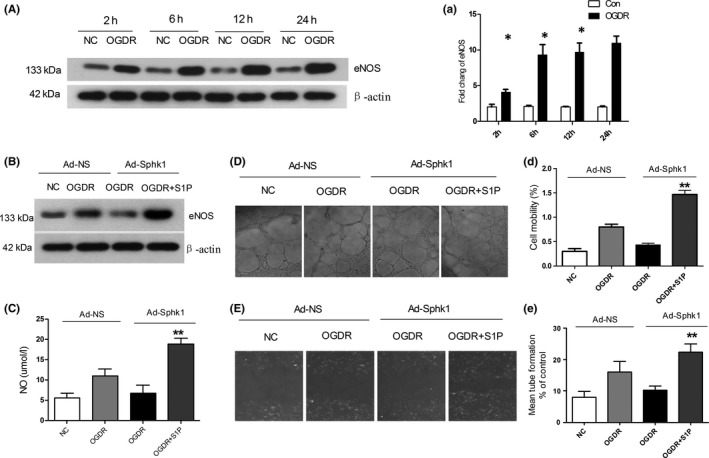
Effects of Sphk1/S1P on HBMEC eNOS induction, migration, and tube formation under OGDR conditions. A, HBMECs were treated under OGDR conditions, and then, eNOS expression was measured by Western blotting with β‐actin used for normalization. B, Following a 6 h OGDR treatment, HBMECs expressing an Sphk1‐specific shRNA were treated using 10 nmol/L S1P for 24 h, and then, eNOS levels were assessed by Western blotting with β‐actin for normalization. C, NO production in cells treated as in (B) was measured. D, Tube formation activity of HBMECs treated as in (B) was assessed in a Matrigel system. E, Cells treated as in (B) were assessed for adhesion migration activity. Data are means ± SD. *P < .05; **P < .01 vs control

We next used an OGDR approach to mimic cerebral IRI conditions in vitro and then examined NOS activity in HBMECs. We found that OGDR conditions rapidly induced eNOS within 6 hours at high levels until 48 hours after treatment (Figure 7A,a). We then knocked down Sphk1 in these HBMECs for 24 hours and then subjected these cells to OGDR conditions for 24. In this context, Sphk1 knockdown led to reduced eNOS induction that could be reversed by exogenous addition of S1P to these cells (Figure 7B). We observed comparable results with respect to NO production (*P* < .01; Figure 7C). We then conducted tube formation and migration assays to assess how Sphk1 might regulate angiogenesis in the context of OGDR (Figure 7D,d,E,e). We observed reduced tube formation by cells in which Sphk1 had been knocked down relative to cells treated with a control lentivirus under OGDR conditions, and exogenous S1P administration was able to reverse this phenotype (*P* < .01; Figure 7D,d,E,e). This indicates that Sphk1/S1P signaling plays a key role in regulating eNOS induction and angiogenesis in this in vitro OGDR model.

## 4 DISCUSSION

Sphk1‐mediated S1P production is known to be an essential mediator of microglial proinflammatory cytokine production.^15^ Sphk1/S1P signaling has previously been shown to pathway regulates microglial IL‐17A production via TRAF2/NF‐κB singling in microglial cells, thereby inducing apoptotic death of neurons in the context of IRI.^15^, ^16^ Sphk1 expression in the brain is basally largely restricted to hippocampal neurons, astrocytes, microglia, and cerebellar granule cells,^29^, ^37^, ^38^, ^39^, ^40^ although previous work by Gao et al have similarly shown expression in HUVEC cells.^25^ Herein, we observed inducible Sphk1 expression in endothelial cells in the context of IRI (Figure 1), and we also found that there are many epithelial cells negative Sphk1 in cerebral ischemia cortex. We inferred that these Sphk1 were produced by microglia or a small number of neurons, which is consistent with our previous experiments.^16^, ^17^ Inhibition of Sphk1 markedly reduced eNOS and iNOS induction in the brain, in addition to reducing neovascularization density relative to control animals (Figures 2 and 3), suggesting that Sphk1 activity is essential for regulating angiogenesis in the context of IRI.

S1P has been shown to be involved in the vascular tube formation process, with roles in regulating vascular maturation 41 and permeability.^42^ In this experiment, we found that exogenous S1P increased eNOS and iNOS in the Ad‐SphK1 group and increased the levels of NO in brain tissue (Figure 4), which indicated that SphK1/S1P may influence angiogenesis and neuroinflammation in the context of cerebral ischemia‐reperfusion injury.

Mice lacking eNOS expression have previously been shown to exhibit a larger infarcted area in the brain following ischemia relative to wild‐type controls,^43^ with eNOS also being known to be essential as a regulator of cell proliferation and apoptosis.^44^, ^45^ In this study, we found that endothelial cells rapidly induced eNOS following cerebral IRI (Figure 2), with Sphk1/S1P being essential mediators of NO generation and angiogenesis in an eNOS‐dependent manner in an HBMEC model of OGDR (Figures 5, 6, 7). Based on this result, we believe that eNOS activity is essential for NO release from endothelial cells, resulting in vasodilation and promoting vascular remodeling following cerebral IRI, which is consistent with previous studies.^46^

We observed a significant number of nNOS‐negative endothelial cells in our model of cerebral IRI, suggesting nNOS production is primarily restricted to neurons. Previous work has shown that in a model of hypoxic‐ischemia, nNOS inhibition was able to reduce brain damage via increasing collateral flow and decreasing the mean blood flow velocities level in the basilar trunk.^47^ In this study, we did not identify a role for Sphk1 in nNOS regulation (Figure 6), suggesting Sphk1 does not play a relevant role in regulating nNOS activity in this model.

iNOS is mainly expressed in microglia, astrocytes, endothelial cells, infiltrating lymphocytes, and macrophages. We were able to detect substantial iNOS induction by later time points following cerebral IRI (Figure 2), and Sphk1/S1P signaling were able to alter this induction, although iNOS did not alter NO production (Figure 6). Following cellular stimulation with inflammatory cytokines, iNOS can be rapidly induced to mediate NO production,^48^ and iNOS inhibition is known to reduced infarct size following cerebral ischemia, thus reducing the associated brain damage.^49^, ^50^ Our results suggest that Sphk1/S1P may play a role in regulating iNOS activity after IRI, potentially altering tissue inflammation without promoting angiogenesis.

In summary, our results offer novel evidence that endothelial Sphk1/S1P signaling regulates brain injury in the context of ischemic stroke in an eNOS‐ and NO‐dependent manner, suggesting that targeting this signaling axis may represent a viable therapeutic strategy for future treatment efforts.

## CONFLICT OF INTEREST

The authors declare no conflict of interest.
