# Supplementary material for: The Sphkl/SlP pathway regulates angiogenesis via NOS/NO synthesis following cerebral ischemia‐reperfusion
Source: CNS Neurosci Ther. 2019 Dec 8;26(5):538–48. doi: 10.1111/cns.13275 (PMC7163582; doi:10.1111/cns.13275)
Supplement: Supplementary file 1 [file CNS-26-538-s001.docx]

[Supplemental Figure](https://fanyi.baidu.com/#en/zh/supplemental%20figure) 1

Rats were injected i.p different concentrations of S1P (0.05 mg/kg, 0.08 mg/kg, 0.1 mg/kg), with the remaining control animals receiving an equivalent saline vehicle injection. After 24 levels of S1P in brain tissue were measured via ELISA. Data are means ± SD. *p < 0.05 vs. control. **P < 0.01, vs. Sham group.
